# Why are toilets not used? Using system effects modelling to understand stakeholder perceptions on the impacts and barriers to *Taenia solium* control in Eastern and Western Uganda

**DOI:** 10.1186/s12917-025-05026-x

**Published:** 2025-10-08

**Authors:** Nicholas Ngwili, Salaviriuse Ahimbisibwe, Derrick N. Sentamu, Luke Craven, Lian F. Thomas, Kristina Roesel

**Affiliations:** 1 Health program, International Livestock Research Institute, Nairobi, Kenya; 2 Department of Public Health Pharmacology and Toxicology, Faculty of Veterinary Sciences, University of Nairobi, Nairobi, Kenya; 3 Partnerships for Local Action and Community Empowerment, Melbourne, Australia; 4 Royal (Dick) School of Veterinary Studies, University of Edinburgh, Easter Bush Campus, Edinburgh, EH25 9RG UK; 5 Department of Animal Breeding and Husbandry in the Tropics and Subtropics, University of Hohenheim, Garbenstr. 17, 70599 Stuttgart, Germany; 6 College of Veterinary Medicine, Animal Resources and Biosecurity, Makerere University, Kampala, Uganda

**Keywords:** System effects modelling, *Taenia solium*, Porcine cysticercosis, Taeniasis, Neurocysticercosis

## Abstract

*Taenia solium* taeniasis/cysticercosis in humans and pigs remains endemic to Uganda. Although, looking at the lifecycle of the parasite, the risk factors are well known, and many biomedical control options exist – no substantial progress has been made in the eradication of *T. solium* infections in Uganda to date. Contextual factors including socioeconomic, cultural and infrastructural factors, may influence the adoption of interventions. A community-based study using mixed methods and relying on system effects modelling approach was carried out between March and April 2021 in Kamuli district, Eastern Uganda, and Hoima district, Western Uganda. System effects modelling is a non-linear methodology that captures the varied nature of the unique, individually lived experiences and aggregates them to reflect what is experienced at a population level. The aim of the study was to capture individual stakeholder perceptions on the consequences of *T. solium* infections and barriers to practice known control options. Overall, 27 factors were identified by 192 participants as consequences of being infected with neurocysticercosis (NCC). For taeniasis, 35 factors were identified with 700 edges/connections made by the participants. Enlargement of stomach, weight loss, diarrhoea, weakness, and stunted growth were the most important consequences. Although porcine cysticercosis (PCC) seemed to be poorly understood by the participants, 14 factors were identified which included poor pig growth, loss of market for pig/pork, and poor pork quality. The study also identified important barriers hindering the adoption of control practices, including lack of knowledge on transmission, sociocultural factors, and resource constraints. For women, lack of knowledge on the mode of transmission and lack of a toilet in the compound ranked highly as important barriers with a weighted degree of 31 and 21, respectively, meaning they were identified by more participants unlike men who ranked lack of a toilet first with a weighted degree of 39, followed by lack of knowledge at 24. Different barriers are associated with the adoption of *T. solium* control practices among community members, stakeholders, and farmers. Despite efforts to address *T. solium* infections, misconceptions and limited understanding persist among stakeholders, particularly regarding NCC and its associated consequences. The system effects approach supports developing contextualized interventions to help in the control of the diseases associated with this parasite.

**Supplementary Information:**

The online version contains supplementary material available at 10.1186/s12917-025-05026-x.

## Background

*Taenia (T.) solium* is a zoonotic cestode which causes *T. solium* taeniasis in humans as well as cysticercosis in humans and pigs [1]. Humans are the definitive hosts harbouring the adult *T. solium* worm (taeniasis) after consuming undercooked or raw pork infected with cysts. Scavenging pigs are exposed to *T. solium* eggs dispersed in the environment through open defecation by taeniasis carriers, resulting in the larval stage cysts developing in the intermediate porcine host porcine cysticercosis (PCC) [2]. Environmental contamination with *T. solium* eggs in areas with poor sanitation and hygiene provides a pathway for humans, as aberrant hosts [3]. They ingest *T. solium* eggs that hatch into larvae which can migrate to striated/heart muscles and sub-cutaneous tissues human cysticercosis (HCC). The cysts tend to migrate and encyst in the central nervous system including the brain, causing human neurocysticercosis (NCC) [4, 5]. In endemic settings, *T. solium* has been estimated to cause approximately 30% of preventable epilepsy cases [5]. *T. solium* is endemic to Uganda, and transmission is largely attributed to the presence of risk factors across pig rearing regions of the country. These include free ranging of pigs, open defecation of infected humans, weak enforcement of pork inspection, consumption of undercooked pork, weak human and animal health services, poverty, and poor sanitation [6–9]. *T. solium* infections are an exemplary health problem which require a One Health approach to address because the diseases occur at the intersection of human, animal and environmental health and its control requires the integration of the three facets, including the social environment [10, 11].

The World Health Organization of the United Nations (WHO) identified *T. solium* as one of the eradicable neglected tropical diseases (NTD) and called for intensified control of the diseases in hyperendemic areas by 2030 through its NTD roadmap 2021–2030 [12]. Control of the parasite involves change of practices including pig husbandry, pork consumption and preparation, individual and household hygiene which can be improved through health education, pig vaccination and treatment and the treatment of humans using dewormers such as praziquantel. Globally, *T. solium* control strategies including health education are implemented in local settings which have unique sociocultural and economic contexts which can influence adoption and need to be well understood [13]. Although, a toolkit of control strategies exist, no substantial progress towards wide-spread control has been made to date [14, 15]. Designing effective strategies and interventions for *T. solium* control requires a deeper understanding of the contexts and drivers that support maintenance of transmission but also the mitigation of its risk factors within households and communities. Contextual factors including socioeconomic, cultural and infrastructural factors may influence the adoption of the interventions as has been shown in Uganda and Zambia [16–18].

The complexity of human behavior, its drivers, how this is amplified in society, and the interconnections between different variables require innovative study methodologies. System effects (SEM) modelling is a non-linear methodology that captures the varied nature of unique individually lived experiences and further aggregates them to reflect what is experienced at a population level. The integrative approach draws on soft systems methodology, fuzzy cognitive mapping, and graph theory [19]. It can investigate different impacts, barriers, enablers, and perceptions around given issues within a system. SEM can therefore help to understand the drivers of human behavior and how to modify them at an individual and societal level to cause change [19, 20].

The current study uses the SEM to understand the local community’s perceptions regarding barriers towards adoption and utilization of practices intended to control *T. solium*. Based on the “how to prevent the pork tapeworm” poster, the transmission of the parasite can be interrupted by using toilets, keeping pigs confined in pens, vaccinating or treating pigs, meat inspection, cooking pork thoroughly to kill the cysts, washing hands, fruits and vegetables and taking dewormers to kill the tapeworms in humans [21]. The current study focuses on toilet use, pig confinement and pork preparation. The study further sought to understand consequences of *T. solium* infections as perceived by the local community. To be able to understand the complex interconnections, we focused on individual perceptions of the consequences and barriers to adopting recommended practices. This ensured we fully captured the dynamics among individuals that shape behavior and decisions making process on the practices to adopt. This empirical evidence will contribute to a better understanding of why levels of *T. solium* remain high even though biomedically, the parasite appears easy to control, potential risk factors are known, and a set of control options exist.

## Materials and methods

### Study area

The study was conducted between March and April 2021 in Kamuli district, Eastern Uganda, and Hoima district, Western Uganda. The two districts have a high number of pig-rearing households and high demand for pig meat and pig products [22]. The study districts have different cultural, socio-economic, physical and environmental contextual factors which shape the occurrence of *T. solium* infections and its control as reported by Ngwili et al. [8].

### Study design and selection of stakeholders

A community-based, mixed qualitative and quantitative study design relying on system effects approach for data collection and analysis was used. We organized community group meetings based on stakeholder roles in *T. Solium* control as shown in Table 1. The participants were selected from the following stakeholder categories: pig farmers, community leaders, animal health assistants, human health assistants and pig/pork traders. Ten community leaders from each district were randomly selected from a list of 30 village leaders provided by the District Veterinary Officers. For farmers, the participants were randomly selected from a list of pig farmers who participated in the cross-sectional study in 2019 [8] using the “random function” in Excel. The remaining categories were purposively selected across the subcounties in the districts following discussion with the district veterinary officer or district health officer. A maximum of 10 participants per category were invited for the group meeting to ensure social distancing as per COVID-19 pandemic protocols. For the pig farmer stakeholder category separate group meeting were held for men and women. Data collection was facilitated by two enumerators fluent in Runyoro language for Hoima district and Lusoga language in Kamuli district with the support of the lead researcher.

**Table 1:** Stakeholder categories and their description

Stakeholder category	Description/Target person or group	Relevance to T. solium control	Method for data collection & number of group meeting across the districts	Number of participants
Pig farmers	Pig farmers randomly selected from a list of pig farmers from 30 villages	They are responsible for control of the parasite at the intermediate and final host stage by practicing proper hygiene and good pig husbandry.	6 with men 6 with women	n men = 56 n women = 60
Community leaders (Local Council chairman 1)	Selected randomly from villages across 3 sub-counties	They are village leaders and are the link between the national government administration and the community. They are involved in enforcing latrine use and other bylaws within the village.	2	n men = 14 n women = 5
Animal Health Assistants	Purposively invited through the district veterinary officer and were drawn from the different sub-counties in the district.	They oversee meat inspection and promotion of good animal husbandry at sub-county level.	2	n men = 14 n women = 4
Human Health Assistants	Purposively selected and invited through the district health officer and were drawn from the different sub-counties in the district.	They oversee human health activities in a sub-county and act as the heads of level 3 health facilities (the government health facility at the sub-county level	2	n men = 12 n women = 8
Pig/pork traders	Selected by snowballing from different sub-counties within the district starting from the district headquarters. Three traders were picked from each sub-county.	They buy pigs from farmers and operate butcheries and pork joints at the sub-country level where they sell raw and ready-to-eat pork.	2	n = 19

### Data collection

**Fig. 1:**
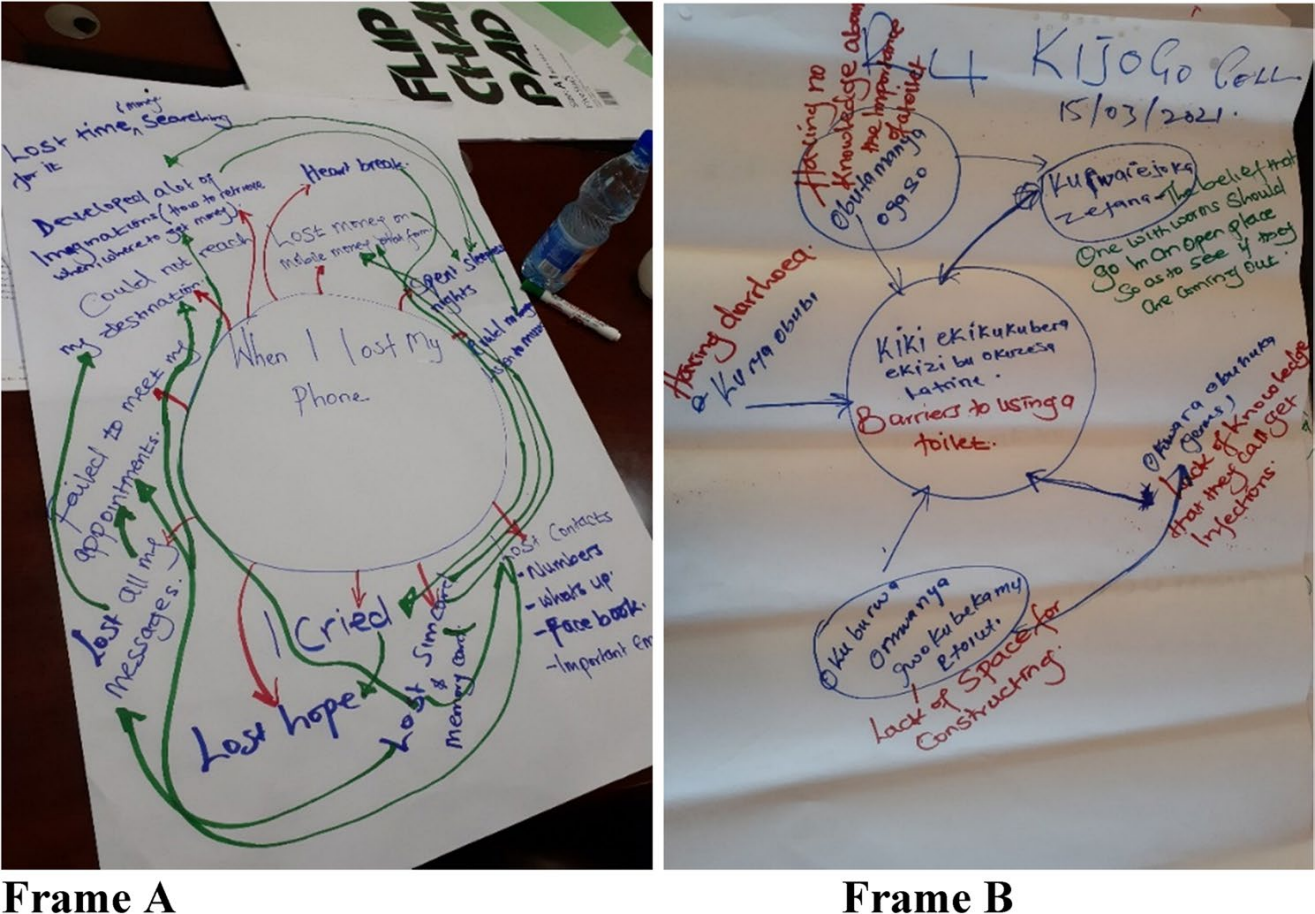
Simulation of capturing consequences of an event under the system effects methodology (Frame A) and capturing barriers to a practice (Frame B). Photo credit: Nicholas Ngwili, ILRI

Participants were invited for a group meeting where *T. solium* infections and the SEM approach was explained to them. This was done to reduce variations in the explanation offered and this was feasible logistically. The data collection was done in two parts focusing on (i) the consequences of being infected with NCC and taeniasis in humans and infection of pigs with PCC and (ii) the barriers to the adoption of practices which can disrupt transmission along the parasite’s life cycle. The participants were issued with flip charts and marker pens to aid in the exercise. To ensure that the participants understood the approach, the process was explained and simulated using a normal occurrence, e.g. the scenario of losing a phone. For consequences, the participants were asked to draw a circle in the middle of the manila paper and write “*when I lost my phone*” in the middle of the circle. They were then guided to individually draw outward-facing arrows to denote the various consequences of losing a phone and draw any connections which existed between the listed consequences to generate a rich picture to relate their experiences and perceptions as described by Craven [19] (Fig. 1).

The simulation exercise was repeated to collect data on barriers to implementing the different practices, with the process being the same, but inward arrows were drawn into the factor/node in the middle of the sketch. The links between the listed factors were drawn to show the interactions between the different factors or nodes within the network. The participants wrote in the local language but at the end of the data collection, the facilitators inserted the English translation with the help of the village elders. Additionally, the facilitators moved around the room and probed for further information on the points listed by the participants. A guide which had developed and reviewed by 2 experts was used which contained the probes the facilitators used to guide the participants through the exercise. Each manila paper was labelled with a unique code representing the participants’ stakeholder category and the village name, photos of each were taken and labelled with the same code. All hard copies were collected and stored.

The participants were asked what makes it hard/difficult or the obstacles in adopting the practices which aim at breaking or disrupting the transmission of the *T. solium* infection. The practices included (1) use of toilets, (2) washing hands, fruits, and vegetables, (3) deworming children and adults regularly, (4) pig confinement, (5) meat inspection, (6) proper cooking of pork, and (7) selling inspected pork. Special focus was given to the farmer category, and the groups are disaggregated by gender. This approach helped us to identify how the different genders experienced the barriers and how they responded to specific challenges affecting them. For the other categories we relied on government officials where gender parity does not exist. The pig or pork trader category was dominated by men and therefore it was not possible to get female participants.

### Data management and analysis

Data entry for both the consequences of and barriers to control the diseases was done in the system effects platform (https://systemeffects.com) developed by Luke Craven. Data were analysed in the same system effects by producing different system effects maps to visualize the relationship and importance between the various nodes. The analysis involved aggregating the individual maps to represent the community. During analysis the individual maps were taken as network diagrams with the factors/consequences representing the nodes and the connection between them taken as edges [23]. Additionally, the data matrix was downloaded as Excel files (.csv), transformed and uploaded as adjacency matrix for further analysis in the network mapping software Gephi (https://gephi.org/) to produce more visuals and statistics. In Gephi, directed network graphs were generated using the Fruchterman-Reingold force directed layout algorithm [24]. The nodes represented the barriers to the identified practices and consequences of the three diseases. Each connection represented the number of participants identifying that link. We had tried to analyse the data at the district level, but this was not producing any reasonable differences, and the factors were too many making visualization difficult hence the difference between the districts was described qualitatively.

We report the PageRank and eigenvector centrality scores for the different nodes which represented the consequences or barriers in the network. Eigenvector centrality shows how a node is central if it is connected to other important nodes within the matrix and it is calculated based on the nodes connection to other nodes and their centrality [25]. On the other hand, PageRank or the importance score is an algorithm that ranks nodes based on their importance by considering the number and quality of inlinks to calculate the importance score [26]. The PageRank score uses the eigenvectors to determine the importance of a node with the matrix or network map. For example, nodes linked to other highly ranked node will have a higher PageRank score. Weighted in-degree is the sum of the weights assigned to each edge/link connecting to a node while weighted out-degree is the sum of the weights assigned to each edge/link connecting from a node [27]. The weighted degree is the sum of both weighted in-degree and out-degree. Betweenness centrality measure the times a node acts as a bridge between two other nodes, in other words the node acting as connectors to other parts of the network [28]. Centrality scores were not because the participants did not build connections between the nodes or factors possibly because they did not comprehend how the points they raised were connected within the system.

For the consequences of the 3 diseases, the participants appeared to have limited knowledge on the transmission pathway and the resultant disease. This led to mix up of the consequences they identified in the sketched network diagram where some consequences were indicated under the wrong infection type. For example, consequences due to PCC being indicated under consequences due to NCC or taeniasis. To make sense of the network, a data cleaning exercise was incorporated where some consequences were dropped during further analysis based on the authors biomedical knowledge and literature.

## Results

Although data were collected for all perceived barriers to toilet use, washing hands, fruits and vegetables, deworming, confinement of pigs, meat inspections, cooking pork well, in this paper, we report on barriers to toilet use, pig confinement and proper pork preparation. These were selected based on the pathway of infections in the *T. solium* life cycle. Infective materials become available in the environment through failure to use toilets or poor sanitation. The life cycle of the parasite is perpetuated by having unconfined pigs which eat the eggs while scavenging and developing the intermediate stage which is infective to humans if poor cooked pork is consumed. Cooking pork well is the last “line of defense” to the humans from infections and therefore barriers to cooking pork well are also emphasized. Moreover, humans ingesting infective eggs from the environment due to poor hand washing practices, may develop NCC as accidental hosts. Additionally, the perceptions on the consequences of taeniasis, NCC and PCC, are presented and discussed. Additional data on barriers to the other practices are mentioned in the tables and figures included in the supplementary materials (supplementary File 1).

### Demographic characteristics

A total of 192 discussants participated in 22 group meetings that comprised of 12 group meetings with pig farmers, two each with animal health assistants, human health assistants, community leaders, and pig/pork traders (Table 1). For the pig farmers, six group meetings were conducted in each district, and one meeting in each district for the other stakeholder categories. The overall sample comprised of 60 women and 56 men for the pig farmer category and 76 participants from the other stakeholder categories all together.

### Consequences of NCC

**Fig. 2:**
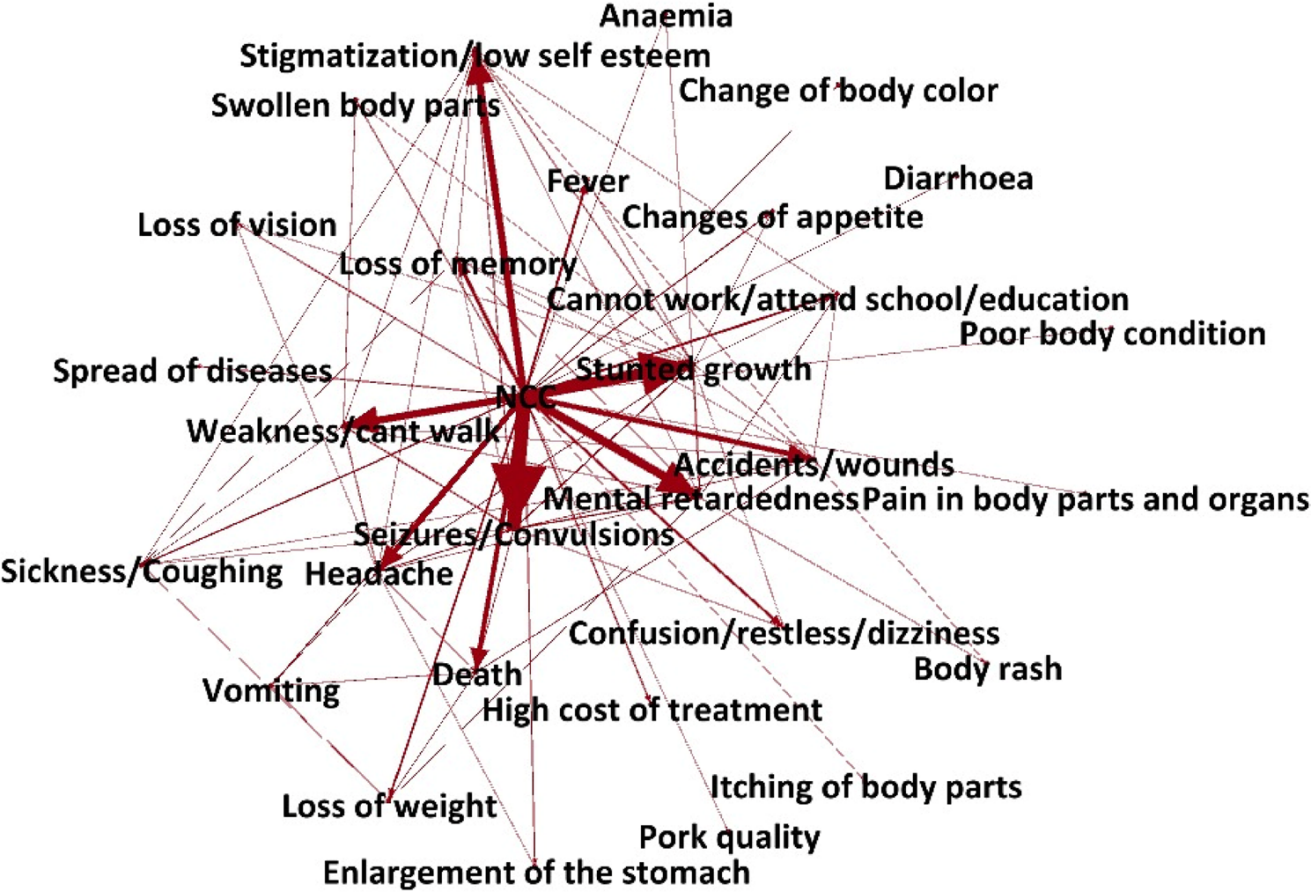
An aggregated network map on the consequences of NCC infections as indicated by the participants. The thickness of the edges (arrows) is proportional to the weighted-out degree representing the number of participants mentioning that node/factor (generated in Gephi)

**Fig. 3:**
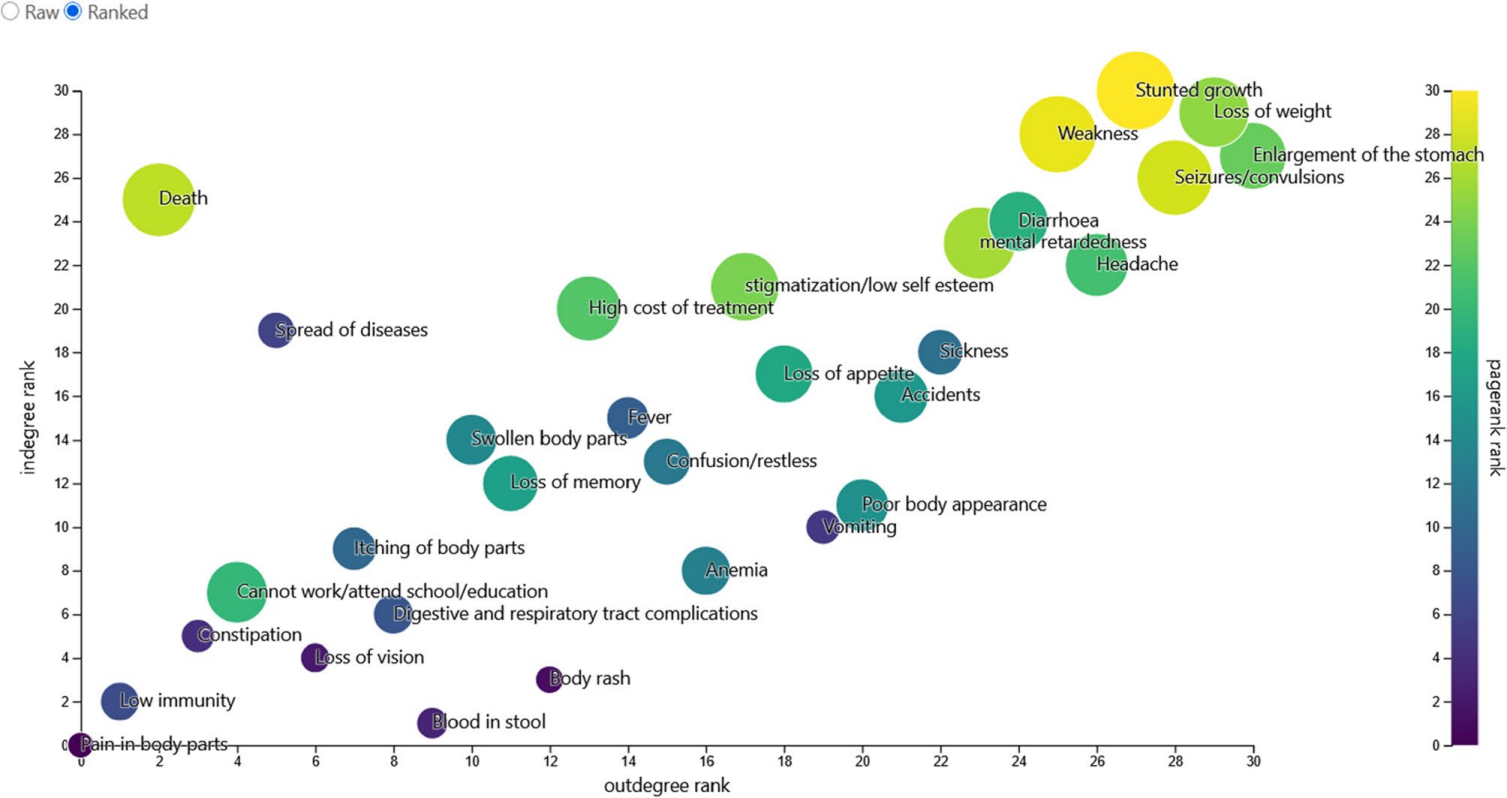
Ranking of consequences of NCC as indicated by the participants by in-degree and outdegree. The circles size represents the PageRank value for each factor (generated in the system effects platform)

Overall, 27 factors were identified by 192 participants as consequences of being infected with NCC. A total of 13 factors were dropped because they appeared to be related to taeniasis and were identified possibly due to lack of knowledge on *T. solium* infections among stakeholders, leaving 14 factors that the participants perceived as direct consequences of NCC. The consequences have been presented in Table 2 and are ranked in descending order based on the number of times they were mentioned by the participants or the weighted degree. The network was also visualized using an aggregated network map with the thickness of the arrows representing the weight of the edges (Fig. 2). Having seizures was mentioned by most participants as shown by the weighted indegree of 121, it was also the most connected node within the network as shown by the weighted outdegree of 36. The importance and influence of having seizures as a node within the network was also high, PageRank of 0.11 and eigen centrality of 1. Stigmatization, inability to attend school, impaired cognitive development, accidents, and death were also influential factors within the network. The participants explained that once someone starts having seizures, nobody wants to associate with them and they cannot work anymore and for school children, they stop going to school. Some nodes also had also acted as intermediaries or bridges by connecting other nodes within the network as shown by the high betweenness centrality. Stunted growth, seizures and headaches had the highest betweenness centrality of 117, 67.1 and 60.5, respectively. When probed, the participants also cited these as the main symptoms of epilepsy. They explained that having epilepsy also affected the growth rate of children and therefore stunted growth was a major consequence. A graphical presentation of the PageRank is presented in Fig. 3 with the size of the circles showing the PageRank of each factor, the larger the circle, the more important the node (high PageRank).

**Table 2:** Network statistics on consequences of infection with NCC as indicated by the participants ranked by importance (weighted degree)

Consequences of NCC infection	Weighted indegree	Weighted outdegree	Weighted degree	PageRank	Eigenvector centrality	Betweenness centrality
Stunted growth	104	21	125	0.157544	0.886571	117
Seizures or convulsions	121	36	157	0.113108	1	67.1
Headache	45	19	64	0.039457	0.363657	60.5
Body weakness	64	17	81	0.053182	0.693327	40.1
Accidents	41	8	49	0.043872	0.385846	25
Delayed mental development	80	15	95	0.060278	0.78063	18.1
Stigmatization	63	4	67	0.066703	0.917229	9.4
Sickness	11	4	15	0.033259	0.419911	5.4
Loss of memory	26	2	28	0.04468	0.504508	5.3
Cannot work/attend school/education	19	1	20	0.05249	0.60088	3
Loss of weight	15	2	17	0.028281	0.261609	1
Death	44	0	44	0.05903	0.432458	0
Confusion	18	1	19	0.035451	0.382157	0
Fever	15	2	17	0.009903	0.001998	0

### Consequences of taeniasis

For taeniasis, 700 edges were made by the participants from 35 factors with 13 of them being dropped because they were not related to taeniasis. Enlargement of stomach, weight loss, diarrhoea, weakness, and stunted growth were the most important consequences of taeniasis, weighted degree of 190, 135, 121, 85 and 74, respectively (Table 3). These factors also appeared to be more influential within the network as shown by the high eigenvector centralities. The network seemed to be more complex and had several nodes being connectors within the network. Enlargement of the stomach, loss of weight, stunted growth, weakness and diarrhoea ranked highly (Table 3). The participants explained that children who had worms usually have large stomachs, do not feed well and hence do not grow well. This shows these nodes are crucial in the flow of information within the network.

**Table 3:** Consequences of taeniasis as indicated by the participants ranked by importance (weighted degree)

Consequences of taeniasis	Weighted indegree	Weighted outdegree	Weighted degree	PageRank	Eigenvector centrality	Betweenness centrality
Enlargement of the stomach	133	57	190	0.076174	0.97119	249.1
Loss of weight	100	35	135	0.067141	0.997176	183.3
Stunted growth	66	8	74	0.128229	0.987568	174.4
Weakness or can’t walk	75	10	85	0.074405	1	111.8
Diarrhoea	97	24	121	0.026123	0.59182	105.5
Itching of body parts	24	1	25	0.024862	0.085243	30.2
Swollen body parts	25	2	27	0.025435	0.431519	29.7
Blood in stool	5	2	7	0.009306	0.111157	25
Anemia	22	4	26	0.02678	0.665298	24.5
High treatment cost	31	1	32	0.064638	0.756681	24.2
Headache	40	14	54	0.020581	0.534601	14.4
Loss of appetite	32	4	36	0.02678	0.665298	11.2
Spread of diseases	5	1	6	0.011553	0.024825	3.8
Shed worms	14	4	18	0.013354	0.292586	0.5
Vomiting	15	2	17	0.011646	0.183303	0.22
Appearing sickly	16	1	17	0.009306	0.111157	0
Constipation	11	1	12	0.011646	0.183303	0
Digestive tract complications	9	2	11	0.007599	0.001873	0
Reduced immunity	5	0	5	0.03016	0.374148	0
Body rash	5	1	6	0.007599	0.001873	0
Pain in body parts	4	0	4	0.007599	0.001873	0
Cannot work/attend school	3	0	3	0.007599	0.001873	0

### Consequences of porcine cysticercosis

For PCC 34 factors were identified by the participants with 20 factors being dropped. Although PCC seemed to be poorly understood, poor pig growth, loss of market for pork, poor pork quality, and spread of diseases were the most identified issues by the participants with weighted degree of 96, 62, 41, 41 respectively (Table 4). Poor body condition, stunted growth and poor pork quality were important connectors or bridges across different parts of the network as shown by the betweenness centrality of 26.5, 18.1 and 11.3, respectively.

**Table 4:** Consequences of PCC in pigs as indicated by the participants ranked by importance (weighted degree)

Consequences of porcine cysticercosis	Weighted indegree	Weighted outdegree	Weighted Degree	PageRank	Eigen centrality	Betweenness centrality
Poor body appearance	19	7	26	0.03776	0.2178	26.5
Stunted growth	85	11	96	0.0392	0.41075	18.1
Poor pork quality	39	2	41	0.09214	0.49467	11.3
Increased cost of treatment cost	26	2	28	0.07555	0.58502	11
Loss of market for pork	60	2	62	0.07244	0.4999	2.5
Spread of diseases	41	0	41	0.01173	0.00392	0
Rough hair coat	32	1	33	0.02897	0.44987	0
Transmits to people	18	6	24	0.01173	0.00392	0
Cysts in tongue	10	2	12	0.01173	0.00392	0
Digestive tract complications	6	0	6	0.02669	0.02195	0
Condemnation of pigs or pork	4	0	4	0.01173	0.00392	0
Change of body color	2	2	4	0.01173	0.00392	0
Reduced immunity	2	0	2	0.01173	0.00392	0
Constipation	2	0	2	0.01173	0.00392	0

### Barriers to toilet use

**Fig. 4:**
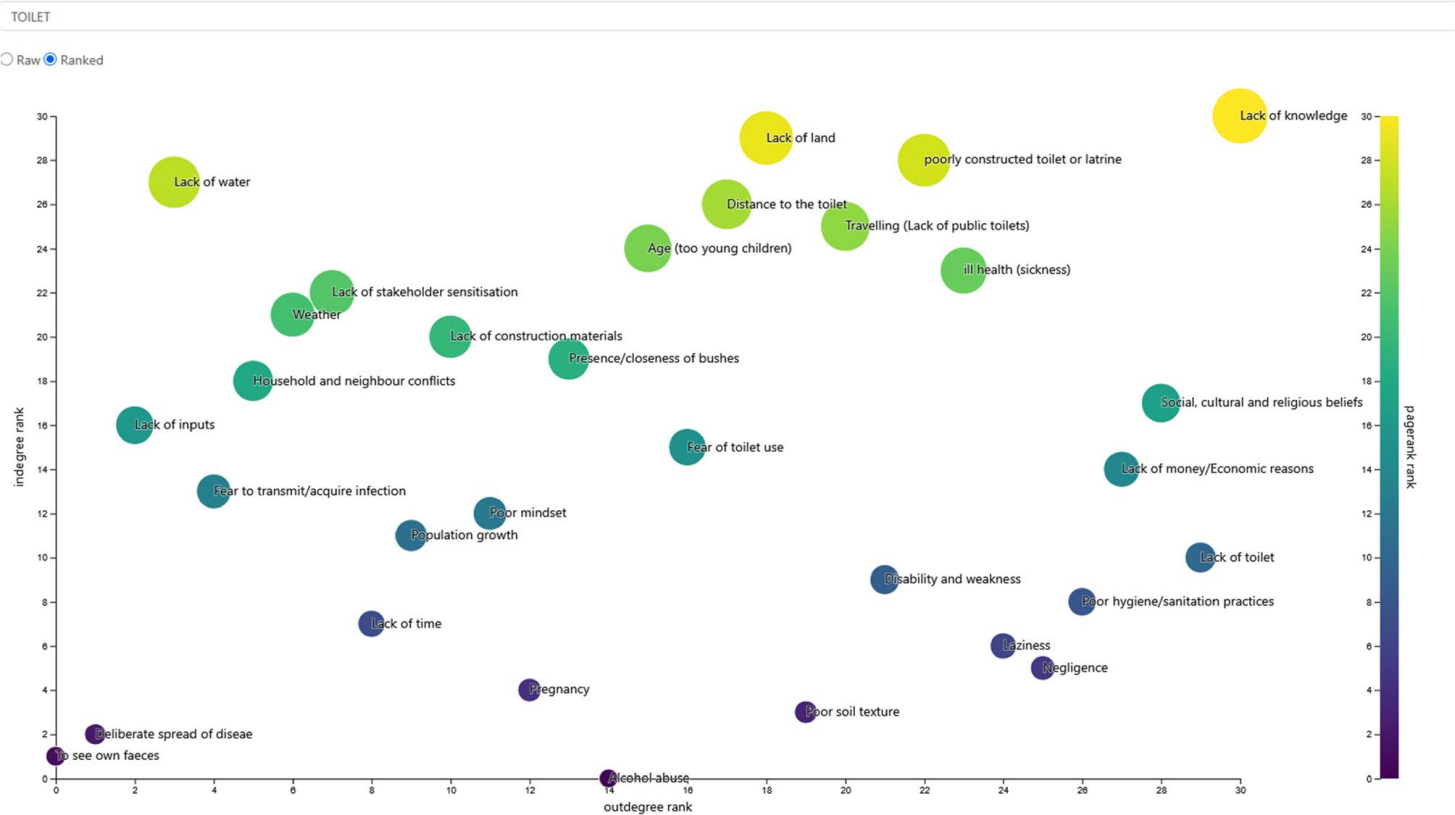
Ranking of barriers to toilet use as indicated by the participants based on in-degree and out-degree with the circles size and colour representing the PageRank value for each factor (generated in system effects platform)

**Fig. 5:**
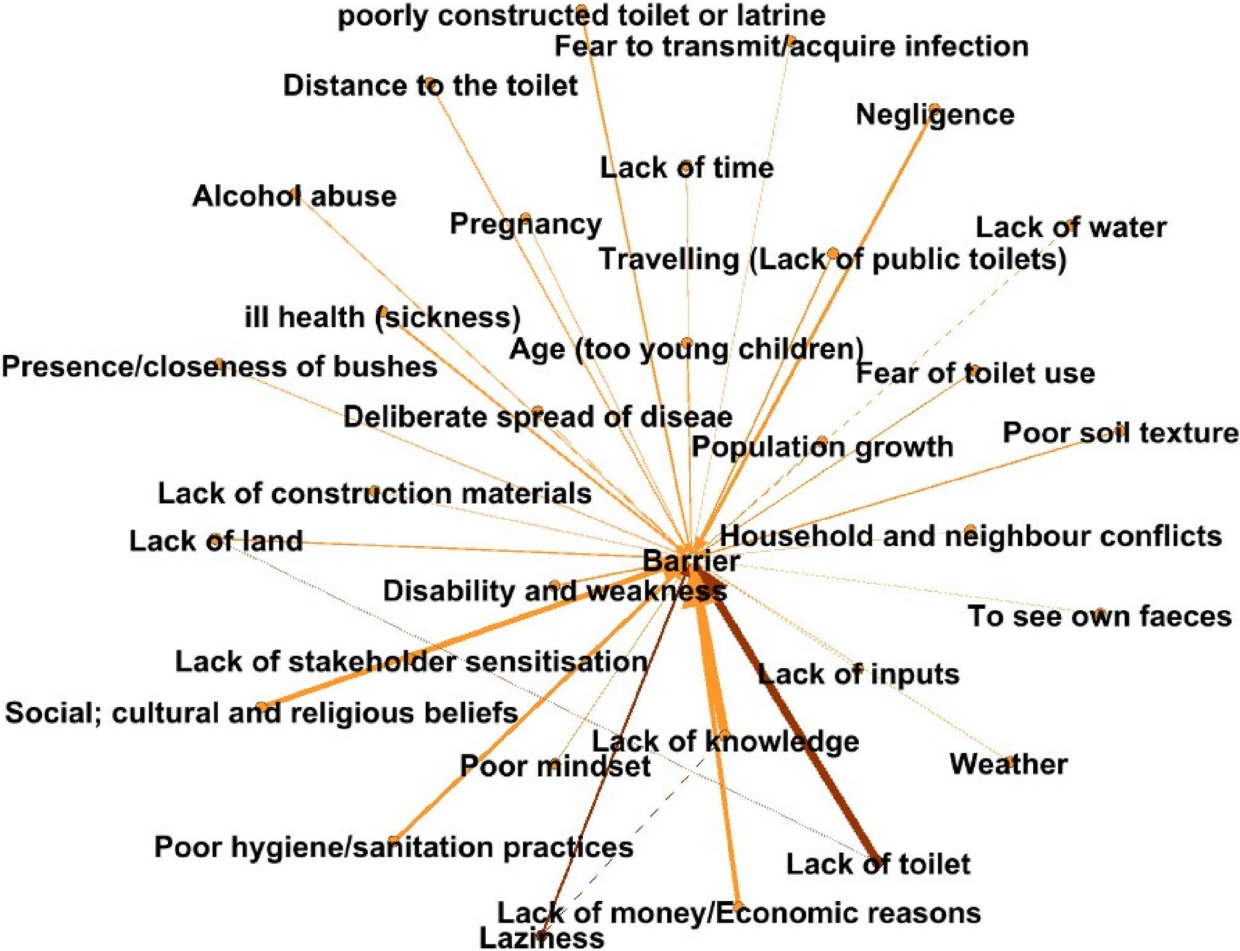
An aggregated network map on the barriers to toilet use as indicated by the participants. The thickness of the edges is proportional to the weighted-out degree representing the number of participants mentioning that node/factor (generated in system effects platform)

Overall, 582 edges were made by the participants from across all stakeholder categories on what makes it hard for people to access and use of toilets. The analysis shows that about 25 factors hindered the use of toilets with 16 of these being identified by majority of the stakeholders (weighted degree > 10) (Table 5). Lack of knowledge on the negative effects of open defecation (97), limited access to toilets (84), sociocultural factors, for example beliefs that pregnant women should not use toilets, women in childbearing age should not use toilets and children faeces are not harmful and therefore they can defecate in the open (55), insufficient funds for construction (48) ranked highly based on the weighted degree. Lack of land and lack of knowledge on toilet construction appeared to be influential and important both having an eigenvector centrality of 0.027 and PageRank of 0.024. This is further supported by the figures visualizing their position and influence within the network map (Figs. 4 and 5). Looking at the difference in the factors across the different stakeholder categories, lack of knowledge was ranked highly by community members (55), community health workers (17) and community leaders (5). The other factors were ranked differently depending on the type of stakeholder with the community members ranking lack of toilets highly at 60, community leaders ranked lack of knowledge at 5 and community health workers ranked sociocultural (e.g. belief that pregnant women should not use toilet) at 18 (see Tables 8, 9 and 10 in supplementary materials).

**Table 5:** Barriers to toilet use as indicated by the participants (aggregated for all stakeholder categories)

Barriers to toilet use	Weighted indegree	Weighted outdegree	Weighted degree	Eigenvector centrality	PageRank
Lack of knowledge	1	96	97	0.027143	0.024126
Lack of toilet	0	84	84	0	0.016934
Social and cultural factors	0	55	55	0	0.016934
Lack of money/Economic reasons	0	48	48	0	0.016934
Negligence	0	34	34	0	0.016934
Laziness	0	27	27	0	0.016934
Ill health (sickness)	0	23	23	0	0.016934
Poorly constructed toilet	0	20	20	0	0.016934
Disability and weakness	0	19	19	0	0.016934
Poor soil texture	0	17	17	0	0.016934
Lack of public toilets	0	17	17	0	0.016934
Lack of land	1	15	16	0.027143	0.024126
Distance to the toilet	0	13	13	0	0.016934
Fear of toilet use	0	11	11	0	0.016934
Alcohol abuse	0	10	10	0	0.016934
Age (too young children)	0	10	10	0	0.016934
Presence/closeness of bushes	0	8	8	0	0.016934
Pregnancy	0	6	6	0	0.016934
Poor mindset	0	5	5	0	0.016934
Population growth	0	4	4	0	0.016934
Lack of construction materials	0	4	4	0	0.016934
Weather	0	3	3	0	0.016934
Lack of stakeholder sensitization	0	3	3	0	0.016934

The data collection and analysis were also disaggregated by gender for the barriers to toilet use because women and men may be affected differently and at varying scales by the factors. There was similarity in the factors identified by both women and men as barriers to toilet use but these factors received different weights. For women, lack of knowledge and lack of toilet in the compound ranked highly as important barriers with a weighted degree of 31 and 21, respectively (Table 6) unlike men who ranked lack of toilet first with a weighted degree of 39, followed by lack of knowledge at 24 (Table 7). Negligence (not willing to construct or use toilet), laziness (not willing to dig and construct toilets), economic reasons (lack of money to construct toilets), and sociocultural were other important factors for both men and women as well as distance to toilets and poorly constructed toilets.

**Table 6:** Barriers to toilet use as identified by female community members

Barriers to toilet use	Weighted outdegree	Weighted Degree	PageRank
Lack of knowledge	31	31	0.02397
Lack of toilet	21	21	0.02397
Negligence	10	10	0.02397
Laziness	10	10	0.02397
Lack of money	10	10	0.02397
Social and cultural factors	10	10	0.02397
Disability	9	9	0.02397
Lack of public toilets	7	7	0.02397
Poor hygiene	6	6	0.02397
Alcohol abuse by men	5	5	0.02397
Ill health	5	5	0.02397
Poorly constructed toilet	5	5	0.02397
Poor soil texture	4	4	0.02397
Lack of land	4	4	0.02397
Fear of toilet use	4	4	0.02397
Age (too young children)	4	4	0.02397
Distance to the toilet	4	4	0.02397
Weather	3	3	0.02397
Household and neighbor conflicts	2	2	0.02397
Pregnancy	1	1	0.02397
Presence of bushes	1	1	0.02397

**Table 7:** Barriers to toilet use as identified by male community members

Barriers to toilet use	weighted outdegree	Weighted Degree	PageRank
Lack of toilet	39	39	0.02397
Lack of knowledge	24	24	0.02397
Poor hygienic practices	17	17	0.02397
Negligence	16	16	0.02397
Ill health	15	15	0.02397
Lack of money	11	11	0.02397
Laziness	9	9	0.02397
Social and cultural reasons	9	9	0.02397
Disability and weakness	6	6	0.02397
Pregnancy	5	5	0.02397
Fear of toilet use	5	5	0.02397
Age	5	5	0.02397
Lack of public toilets	5	5	0.02397
Distance to the toilet	4	4	0.02397
Poorly constructed toilet or latrine	4	4	0.02397
Alcohol abuse	2	2	0.02397
Lack of land	2	2	0.02397
Lack of time	2	2	0.02397
Presence of bushes	2	2	0.02397
Need to see own faeces	1	1	0.02397
Fear of getting infection	1	1	0.02397
Lack of water for use in toilets	1	1	0.02397

### Barriers to proper cooking of pork

The most important barriers as indicated by their weighted degrees were lack of time to cook pork well (92), lack of fuel or firewood (79), liking for meat (impatience to wait until it is ready) (78), and lack of knowledge on how to prepare the pork (Table 4 in supplementary material). Other factors include the preference for meat, having too many clients or users who pressure the pork joint operator to cook fast, extreme hunger, lack of cooking skills, negligence (not willing to do the right thing), lack of cooking utensils, laziness, alcohol abuse, profit maximization (interested in selling more than ensuring pork is well cooked), and lack of water.

### Barriers to pork inspection

Based on our analyses, there were several significant obstacles to inspecting pork for human consumption as shown by the high weighted degree. These included the lack of centralized slaughter points (33), knowledge gaps in how to conduct inspection of pork (30), low staffing of meat inspectors (16), lack of laws to support enforcement of meat inspection (10) as well as resistance from some actors and interference from other state or political actors (15). Other barriers included the lack of inspection tool kits (11), and corruption (9) (Table 5 in supplementary materials). Moreover, there were traders who avoided inspectors, lack of stakeholder sensitization, transport for inspectors, and sick pigs being cheaper and therefore traders bought these intentionally to save on cost. Additionally, there were difficulties in detecting cysts by the inspectors, and other barriers such as lack of collaboration between stakeholders, insecurity (the meat inspectors feeling not safe from physical harm or threat as they enforce meat inspection regulations),, negligence, poor mindset (not interested in doing the right thing), laziness, failure to compensate the pig owner for condemning the meat, alcohol abuse (consumer not able to inspect pork when buying), poor accessibility to slaughter points with skilled meat inspectors, inapparent infection (refers to PCC being asymptomatic), and the effect of cysticercosis not being appreciated by the stakeholders.

### Barriers to washing hands, fruits, and vegetables

The key barriers to adequate washing of hands and fruits included lack of knowledge on why it is important to wash hands and fruits (91), lack of water (85) lack of time to wash hands or fruits before eating (35), liking for meat making people impatient (11) (do not wash hands before eating), laziness (46), lack of washing facilities (42), poor hygiene and sanitation practices (like not using soap) (28), negative mindset (not willing to do the right thing) (7), disability and weakness (5).(Table 6 in supplementary materials).

### Barriers to pig confinement

Farmers faced a range of challenges confining their pigs. These obstacles were financial due to a lack of resources to construct pig pens (35), lack of knowledge (27), or lack of regulations and their enforcement (6). Other factors impede farmers from confining their pigs include inadequate facilities or land, neglect, resistance from some stakeholders, the desire to allow free-range grazing due to the believe that it will improve growth rates, boar service, and a preference for low-input production systems (Table 7 in supplementary materials).

## Discussion

System effects modelling approach of facilitating group meetings captures the varied nature of individual’s lived experiences. These individual responses are then aggregated to produce a map which helps understand the problem better by considering all individual experiences. In the current study the SE approach was used to gain a deeper understanding of the consequences of the three *T. solium* diseases, and the barriers individuals experience in adopting practices which can help control infections with the parasite. Although many qualitative studies on knowledge, attitudes and perception of *T. solium* infections have relied on consensus building through focus group discussions, we chose to build individual maps to maximise on the number of items or factors the individuals generated. Guest et al. [29] in a randomized study to compare group versus individual based data collection in qualitative studies, noted that individual interviews were better in generating a broad range of items. Additionally, individual interviews have been found to be better in bringing out respondent’s personal thoughts, feelings, and world view. For the consequences of the 3 diseases, the participants appeared to have limited knowledge on the transmission pathway and the resultant disease. This led to mix up of the consequences they identified in the sketched network diagram where some consequences were indicated under the wrong infection type. For example, consequences due to PCC being indicated under consequences due to NCC or taeniasis. This was expected among lay people because the infection of pigs with PCC remains asymptomatic over the infection period except the infection of the brain tissues with cysts which may produce some neurological symptoms.

The factors influencing the ability of community members, stakeholders, and farmers to adopt *T. solium* control practices operate at different levels including national and at community level or household level and affect individuals differently. These factors also operate under a complex ecosystem of factors and contextual background with a lot of uncertainty as has been observed in other global challenges such as food insecurity [19, 30, 31]. There are also connections to the broader socioeconomic and political settings and influences which shape how people perceive problems affecting them and how to respond to them. In the current study, the use of exploratory approaches to build network models as described by Hevey [32] to understand the complexities and uncertainties which shape the perception of *T. solium* infections and its control was helpful.

The stakeholders appeared to have a clearer understanding of NCC compared to taeniasis although they mostly concentrated on consequences associated with epilepsy which may or may not be caused by the pork tapeworm. Having seizures was the most named consequence of having NCC and the most important and more influential node in the network. Psychological studies based on the Illness Perception Questionnaire Revised (IPQ-R) have shown that illness perception is based on five attitudinal dimensions which include illness identity, cause, timeline about the duration of the illness, consequence, control and illness coherence [29]. We believe that these mental representations of illness, which are composed of parallel cognitive, emotional dimensions shaping illness-related behaviors came to play when the participants were responding to the questions regarding taeniasis and NCC [33, 34]. This further supports our choice of building the network maps at individual level.

One of the major characteristics of epileptic patients regardless of the cause of the infection is having seizures but this is not the only symptom of epilepsy as this varies from patient to patient with some experiencing confusion and severe headache [35–37]. Consequences due to the seizures such as accidents, delayed mental development and stigmatization were identified by the participants. The burden of epilepsy has been estimated for Uganda and Tanzania and other regions of the world and globally. For Uganda was estimated at 170,000 disability-adjusted life years (DALYs), in Tanzania 3.0 per 1000 person-years and 2.8 million DALYs globally [35, 38, 39]. The fact that the participants perceived the consequences of epilepsy highly may form a good basis for pushing stakeholders to adopt *T. solium* control practices but only if they can clearly understand the link between the infections and NCC which usually is not the case as reported by Ngwili et al. where stakeholders could not link *T. solium* infection in pigs and the infection in humans [18].

Taeniasis was confused with other gastrointestinal infections including round worms (*Ascaris* spp.), whipworms (*Trichuris* spp.), or threadworms or pinworms (*Enterobius* spp.). Therefore, the mention of some of the consequences which can also be attributed to these infections. It is worth noting that most worm infections are similar, and although tapeworm segments can be recognized and look different from *Ascaris* spp. or *Trichuris* spp., the eggs of those worms shed by humans can also infect pigs and lead to chronic disease in pigs (that resemble the signs of sickness that respondents listed like the rough coat, weight loss etc.) as observed previously [8, 10]. The participants’ positive perception of gastrointestinal parasites infections even if not directly on the pork tapeworm is an important indicator which can be leveraged to improve health seeking behaviour. For instance regular deworming, or mass deworming of children, who are the dominant group defecating in the open, with albendazole and mebendazole which are known to be broad spectrum and have been used to treat tapeworm and other gastrointestinal infections [40, 41].

For PCC, the stakeholders have somewhat better perception and understanding of the disease, but three consequences stood out in the network model. Loss of market for pork was possibly due to traders not willing to buy infected pork with cysts and condemnation of pigs by government meat inspection officials at slaughter were important consequences. These indicated some level of understanding about the disease in pigs but not good knowledge on its transmission. This good perception can create good local stakeholder support for interventions aimed at reducing the spread of the disease in pigs PCC is asymptomatic, meaning it does produce noticeable symptoms in pigs, and this makes farmers not perceive it as a major production limiting disease. Similar findings were reported by Ngwili et al. [18] who reported that farmers in Uganda had poor and fragmented knowledge on *Taenia solium* infections. Similarly Zulu et al. in a study in Zambia reported that, although, participants were aware of cysts in pigs they did not know the mode of transmission and prevention [42]. Similar findings have been reported in northern Uganda where Ngwili et al. reported that value chain actors were aware of cysts found in pork but they had low knowledge on the transmission [43]. Overall, for the consequences of taeniasis, NCC and PCC, some have factors had a high betweenness centrality which measures the importance of a node based on the node with the highest frequency of connection along the shortest path [44]. For example, stunted growth had high between centrality in all the three network maps meaning for all the consequences identified, this factor acted as a connector to other consequences. This could imply that the participants tried to understand the diseases better and express their perception more accurately.

The maintenance of the transmission of *T. solium* within a community depends solely on the availability of infective eggs in the environment with the only source being human faeces from a tapeworm carrier. Therefore, understanding what makes it difficult for people in endemic areas to use toilets may contribute to designing better educational interventions to change behaviour. Lack of knowledge was an important factor in the network model; this could have been the lack of knowledge on the negative effects of not using a toilet, or a lack of knowledge and skills to construct toilets. The lack of land to build the toilets was another important factor which was compounded by poor knowledge on toilet construction and lack of resources to construct the toilets. This could be explained by the high levels of poverty and the type of land tenure which is more communal under the Bunyoro and Busoga kingdoms in Hoima and Kamuli districts, respectively, making people view themselves as not having a place of their own to build a permanent toilet. This is further worsened by the type of soil structure which may not support toilet construction because the structure is weak made up of mostly sandy soils near the water basins, or the soils being rocky and on steep slopes making construction difficult and expensive as reported in Ngwili et al. [18].

Farmers were more affected by lack of knowledge on toilet construction while community health workers identified sociocultural factors such as lack of resources to construct toilets as being important barriers facing farmers. Some factors, for example, ignorance, not willing to work hard, alcohol abuse, presence of bushes which make people feel they have enough privacy for open defecation may require innovative interventions aimed at changing human behaviour including the psychological and social drivers [45, 46]. For female community members, the lack of a toilet was an important barrier possibly because they felt that this was beyond their control in terms of the responsibility of ensuring the household has toilet which is a responsibility entrusted to male heads of the family. Toilet construction is both labour intensive and expensive, and in most male headed households the responsibility of the men. Ngwili et al. [18] in the same regions of Uganda reported that the role of men was to dig the pit, cut logs and build the toilet with the women supporting the construction by cutting grass for thatching and fetching water. Similary, in Zambia the role of building toilets belonged to men [16]. Lack of knowedge or ignorance, cleanliness, on the need to use toilets was also an important factor and has also been noted in other studies [16, 18].

Factors that made it hard for participants to cook or roast pork well were lack of time to cook pork well, lack of fuel or firewood, and the liking for meat (unable to wait until it’s ready) as indicated by the network map. These findings highlight the fact that the participants appreciated that sometimes they did not cook pork properly, and that, they ate poorly cooked pork especially when they ate outside the household in butcheries and roadside eateries (pork joints). Proper cooking of pork at 80 °C for at least 10 min has been shown to be sufficiently kill viable *T. solium* cysts, hence eliminating the risk of transmitting the parasite [47]. Eating undercooked pork can lead to a range of zoonotic infections, including taeniasis which results from ingesting viable *T. solium* metacestodes [48]. Eating undercooked pork has been documented in other tropical areas where taeniasis remains endemic, such as Tanzania [49] and Vietnam [50] and LAOs [51]. Although, consumers might have an idea about the advantages of eating properly cooked pork, the barriers identified in this study and in other studies are mainly driven by socioeconomic and cultural factors [49, 52]. This makes it more critical to try to understand the contextual influences in these endemic areas and design interventions aimed at changing human behavior and empowering the different stakeholder categories.

In rural Uganda, pork is not routinely inspected, and even when done, it is done at the butchery or pork joint and not the slaughter facility [43]. The absence of centralized slaughter facilities constitutes a significant impediment to the efficient inspection of pork. Backyard slaughter being the only alternative in the area, implying that the inspector must travel from one pork joint or butchery to another scattered over a larger geographical area. These form part of the barriers identified by the stakeholders and which in most cases were beyond the control of the officials at local level where more focus is given to cattle value chains in budgetary allocation. Similar findings have been reported in other studies in Uganda, for example in northern Uganda pig slaughter occurred in undesignated slaughter points which are rarely visited by meat inspectors [43] and staff absenteeism, insufficient and unpredictable budgets, weak legislation were reported as major challenges affecting the veterinary service delivery in Uganda [53].

A major driver for *T. solium* infections is free roaming pigs especially in communities with poor sanitation where the pigs are able to pick infective materials in the environment as they scavenge for feed [54]. While the participants were aware that free-ranging pigs could potentially expose them to risks such as diseases and worms, they chose to allow them to roam due to their inability to confine them or the need to meet production targets such as weight gain or boar service without incurring costs in terms of feeding and paying for mating services, respectively. Free ranging is still common in other parts of Uganda where landholding is averagely still high coupled with low-resource investments in piggery [55]. In areas where the population density is relatively high and people have small parcels of land; they tend to be pushed to practice some form of confinement to prevent pigs from destroying neighbours’ crops unlike areas with larger parcels of land. Pigs are kept in low-input low-output production systems, often for purposes of meeting immediate livelihood needs such as school fees or medical care, and not for profitability but as a household asset [55]. This temporary production aim coupled with the lack of means for capital investments, contributes to producers choosing low-input systems as observed by Lekule et al. [56] and Thomas et al. [57].

Access to water and sanitary facilities remains a challenge for most communities in low- and middle-income countries (LMICs), although some improvements are being made [58]. As observed in this study, the lack of washing facilities and negligence were highlighted as key barriers to proper hygiene. Furthermore, these barriers are compounded by poverty, characterized by a lack of resources, food insecurity, and low levels of knowledge regarding foodborne illnesses. Community hygiene and household hygiene including washing hands and fresh food like fruits and vegetables can contribute to preventing NCC through the ingestion of the parasite eggs. Although, hand washing behaviour significantly improved during the COVID 19 pandemic [18], barriers still existed preventing complete shift in behaviour. In a review conducted by Tseole et al. [59] in Southern Africa who highlighted similar issues such as ignorance or lack of knowledge, defective local community engagement and poor financing affecting interventions aimed at improving household hygiene.

Unlike other methodological approaches which show linear associations, SEM use complexity frameworks to understand the interrelations of the consequences of the *T. solium* infections and the barriers to the control of the parasite. These consequences and barriers are complex and shaped by individual experiences, perceptions and knowledge. By focusing on the individual perceptions of the consequences and barriers, system effects can capture and help us understand the complex dynamics through which *T. solium* infections are perceived and help amplify contextualized individual experiences within a system which would otherwise be lost within group responses based on consensus or the opinions of the majority [19]. However, some limitations exist including the potential for bias through collection of the data based on self-reported practices and poor understanding of the data collection approach among stakeholders with low levels of education like the rural farmers.

## Conclusions and recommendations

The study highlights the multifaceted barriers associated with the adoption of *T. solium* control practices among community members, district level officials, and farmers. The findings show the interplay of various factors operating at different scales, influenced by complex ecosystem dynamics and contextual backgrounds. Despite efforts to address *T. solium* infections, misconceptions and limited understanding persist among stakeholders, particularly regarding NCC and its associated consequences. While epilepsy is recognized as a significant consequence of *T. solium* infections, its linkage to NCC and *T. solium* infections is not always clear to stakeholders. The study also identifies important barriers hindering the adoption of practices that lead to the control of parasite, including lack of knowledge, sociocultural factors, and resource constraints. Challenges such as absence or inadequate sanitation facilities, poor cooking practices, and weak enforcement of pork inspection contribute to the perpetuation of *T. solium* transmission.

To address these challenges effectively, targeted interventions are needed, considering the unique context of each community. Educational campaigns should prioritize raising awareness about *T. solium* infections, emphasizing the link between pork consumption and disease transmission. Efforts to improve sanitation infrastructure and promote hygienic practices are essential, alongside measures to enhance pork inspection and production standards. Moreover, empowering stakeholders through capacity building and community engagement initiatives can foster sustainable behavior change and enhance control efforts. More broadly this study illustrates the importance of considering social, cultural, and other issues within the discipline of One Health as they make a significant contribution to transmission and impact of NTDs like *T. solium.*

## Supplementary Information

Supplementary Material 1.

## Data Availability

Data is provided within the manuscript or supplementary information files.
