# Supplementary material for: Why are toilets not used? Using system effects modelling to understand stakeholder perceptions on the impacts and barriers to Taenia solium control in Eastern and Western Uganda
Source: BMC Vet Res. 2025 Oct 8;21:588. doi: 10.1186/s12917-025-05026-x (PMC12505678; doi:10.1186/s12917-025-05026-x)
Supplement: Supplementary file 1 — Supplementary Material 1. [file 12917_2025_5026_MOESM1_ESM.docx]

**Supplementary materials: Statistical tables for the consequences of *T. solium* diseases**

Table 1: Consequences of porcine cysticercosis

| **Consequence** | **weighted indegree** | **weighted outdegree** | **Weighted Degree** | **pageranks** | **eigencentrality** |
| --- | --- | --- | --- | --- | --- |
| Death | 59 | 0 | 59 | 0.139101 | 1 |
| Weakness/can't walk | 32 | 1 | 33 | 0.063595 | 0.690919 |
| Loss of weight | 86 | 17 | 103 | 0.096219 | 0.641574 |
| Increased cost/high treatment cost | 26 | 2 | 28 | 0.075552 | 0.585023 |
| Loss of market for pork | 60 | 2 | 62 | 0.072442 | 0.4999 |
| Pork quality | 39 | 2 | 41 | 0.092141 | 0.49467 |
| Pig hair coat | 32 | 1 | 33 | 0.028972 | 0.449865 |
| Stunted growth/poor growth/emaciation | 85 | 11 | 96 | 0.039202 | 0.410746 |
| vomiting | 9 | 1 | 10 | 0.025911 | 0.284896 |
| Poor body appearance/condition/poor herd production | 19 | 7 | 26 | 0.037759 | 0.217798 |
| Enlargement of the stomach | 24 | 2 | 26 | 0.018257 | 0.030257 |
| Digestive and respiratory tract complications | 6 | 0 | 6 | 0.026692 | 0.021949 |
| loss of appetite/increased appetite/eat a lot | 10 | 2 | 12 | 0.018839 | 0.021245 |
| Diarrhea | 28 | 3 | 31 | 0.014226 | 0.012936 |
| stigmatization/low self esteem | 3 | 2 | 5 | 0.016719 | 0.012936 |
| Spread of diseases | 41 | 0 | 41 | 0.011733 | 0.003924 |
| Sickness/Coughing | 24 | 4 | 28 | 0.011733 | 0.003924 |
| Transmits to people | 18 | 6 | 24 | 0.011733 | 0.003924 |
| Cysts in tongue/worms in meat | 10 | 2 | 12 | 0.011733 | 0.003924 |
| Fever | 7 | 0 | 7 | 0.011733 | 0.003924 |
| itching anus/itching of other body parts/irritation | 4 | 1 | 5 | 0.011733 | 0.003924 |
| Body rash | 4 | 1 | 5 | 0.011733 | 0.003924 |
| Swollen body parts | 4 | 0 | 4 | 0.011733 | 0.003924 |
| Condemnation of pigs/pork | 4 | 0 | 4 | 0.011733 | 0.003924 |
| Change of body color | 2 | 2 | 4 | 0.011733 | 0.003924 |
| Confusion/restless/dizziness | 2 | 1 | 3 | 0.011733 | 0.003924 |
| Shed worms | 3 | 0 | 3 | 0.011733 | 0.003924 |
| Anemia/Dehydration/Loss of blood | 2 | 1 | 3 | 0.011733 | 0.003924 |
| Headache | 3 | 0 | 3 | 0.011733 | 0.003924 |
| Blood in stool | 2 | 0 | 2 | 0.011733 | 0.003924 |
| Low/reduced immunity | 2 | 0 | 2 | 0.011733 | 0.003924 |
| Constipation | 2 | 0 | 2 | 0.011733 | 0.003924 |
| seizures/convulsions/paralysis/falling/fainting/loss of conciousness | 1 | 0 | 1 | 0.011733 | 0.003924 |
| Accidents/wounds | 1 | 0 | 1 | 0.011733 | 0.003924 |
| PCC | 0 | 583 | 583 | 0.011447 | 0 |

Table 2: Consequences of Taeniasis

| **Consequences** | **weighted indegree** | **weighted outdegree** | **Weighted Degree** | **pageranks** | **eigencentrality** |
| --- | --- | --- | --- | --- | --- |
| Taeniasis | 0 | 700 | 700 | 0.007428 | 0 |
| loss of vision/ effects on eyes | 6 | 1 | 7 | 0.007599 | 0.001873 |
| itching anus/itching of other body parts/irritation | 24 | 1 | 25 | 0.024862 | 0.085243 |
| Pain in body parts and organs | 4 | 0 | 4 | 0.007599 | 0.001873 |
| Digestive and respiratory tract complications | 9 | 2 | 11 | 0.007599 | 0.001873 |
| Blood in stool | 5 | 2 | 7 | 0.009306 | 0.111157 |
| Low/reduced immunity | 5 | 0 | 5 | 0.03016 | 0.374148 |
| Poor body appearance/condition/poor herd production | 10 | 2 | 12 | 0.011646 | 0.183303 |
| Constipation | 11 | 1 | 12 | 0.011646 | 0.183303 |
| Swollen body parts | 25 | 2 | 27 | 0.025435 | 0.431519 |
| Sickness/Coughing | 16 | 1 | 17 | 0.009306 | 0.111157 |
| Cysts in tongue/worms in meat | 1 | 0 | 1 | 0.007599 | 0.001873 |
| mental retardedness | 12 | 2 | 14 | 0.032449 | 0.207511 |
| Confusion/restless/dizziness | 11 | 2 | 13 | 0.016036 | 0.369823 |
| Loss of memory | 4 | 1 | 5 | 0.007599 | 0.001873 |
| Accidents/wounds | 1 | 0 | 1 | 0.007599 | 0.001873 |
| Enlargement of the stomach | 133 | 57 | 190 | 0.076174 | 0.97119 |
| Body rash | 5 | 1 | 6 | 0.007599 | 0.001873 |
| Change of body color | 14 | 3 | 17 | 0.011989 | 0.188393 |
| Shed worms | 14 | 4 | 18 | 0.013354 | 0.292586 |
| Anemia/Dehydration/Loss of blood | 22 | 4 | 26 | 0.02678 | 0.665298 |
| vomiting | 15 | 2 | 17 | 0.011646 | 0.183303 |
| Stunted growth/poor growth/emaciation | 66 | 8 | 74 | 0.128229 | 0.987568 |
| Increased cost/high treatment cost | 31 | 1 | 32 | 0.064638 | 0.756681 |
| loss of appetite/increased appetite/eat alot | 32 | 4 | 36 | 0.02678 | 0.665298 |
| Fever | 16 | 1 | 17 | 0.039242 | 0.551659 |
| Weakness/cant walk | 75 | 10 | 85 | 0.074405 | 1 |
| Cannot work/attend school/education | 3 | 0 | 3 | 0.007599 | 0.001873 |
| Spread of diseases | 5 | 1 | 6 | 0.011553 | 0.024825 |
| stigmatization/low self esteem | 4 | 0 | 4 | 0.016122 | 0.180608 |
| Death | 35 | 0 | 35 | 0.063774 | 0.813898 |
| Headache | 40 | 14 | 54 | 0.020581 | 0.534601 |
| Diarrhoea | 97 | 24 | 121 | 0.026123 | 0.59182 |
| Loss of weight | 100 | 35 | 135 | 0.067141 | 0.997176 |

Table 3: Consequences of NCC

| **Consequence** | **weighted indegree** | **weighted outdegree** | **Weighted Degree** | **pageranks** | **eigencentrality** |
| --- | --- | --- | --- | --- | --- |
| NCC | 0 | 577 | 577 | 0.009621 | 0 |
| loss of vision/ effects on eyes | 6 | 1 | 7 | 0.014096 | 0.078347 |
| itching anus/itching of other body parts/irritation | 1 | 0 | 1 | 0.009903 | 0.001998 |
| seizures/convulsions/paralysis/falling/fainting/loss of conciousness | 121 | 36 | 157 | 0.113108 | 1 |
| Pain in body parts and organs | 1 | 0 | 1 | 0.009903 | 0.001998 |
| Poor body appearance/condition/poor herd production | 1 | 0 | 1 | 0.009903 | 0.001998 |
| Swollen body parts | 6 | 1 | 7 | 0.016363 | 0.141166 |
| Sickness/Coughing | 11 | 4 | 15 | 0.033259 | 0.419911 |
| mental retardedness | 80 | 15 | 95 | 0.060278 | 0.78063 |
| Confusion/restless/dizziness | 18 | 1 | 19 | 0.035451 | 0.382157 |
| Loss of memory | 26 | 2 | 28 | 0.04468 | 0.504508 |
| Accidents/wounds | 41 | 8 | 49 | 0.043872 | 0.385846 |
| Enlargement of the stomach | 5 | 1 | 6 | 0.009903 | 0.001998 |
| Body rash | 2 | 1 | 3 | 0.009903 | 0.001998 |
| Change of body color | 1 | 0 | 1 | 0.009903 | 0.001998 |
| Anemia/Dehydration/Loss of blood | 2 | 0 | 2 | 0.024775 | 0.183104 |
| vomiting | 6 | 4 | 10 | 0.014096 | 0.078347 |
| Stunted growth/poor growth/emaciation | 104 | 21 | 125 | 0.157544 | 0.886571 |
| Increased cost/high treatment cost | 7 | 0 | 7 | 0.009903 | 0.001998 |
| loss of appetite/increased appetite/eat alot | 7 | 1 | 8 | 0.024775 | 0.183104 |
| Fever | 15 | 2 | 17 | 0.009903 | 0.001998 |
| Weakness/cant walk | 64 | 17 | 81 | 0.053182 | 0.693327 |
| Cannot work/attend school/education | 19 | 1 | 20 | 0.05249 | 0.60088 |
| Spread of diseases | 5 | 0 | 5 | 0.009903 | 0.001998 |
| stigmatization/low self esteem | 63 | 4 | 67 | 0.066703 | 0.917229 |
| Pork quality | 1 | 0 | 1 | 0.009903 | 0.001998 |
| Death | 44 | 0 | 44 | 0.05903 | 0.432458 |
| Headache | 45 | 19 | 64 | 0.039457 | 0.363657 |
| Diarrhoea | 1 | 0 | 1 | 0.009903 | 0.001998 |
| Loss of weight | 15 | 2 | 17 | 0.028281 | 0.261609 |

**Table 4. Barrier to proper cooking of pork**

| Barrier to cooking pork well | Weighted indegree | Weighted outdegree | Weighted degree | Eigenvector centrality | PageRank |
| --- | --- | --- | --- | --- | --- |
| Lack of time | 0 | 92 | 92 | 0.019635 | 0 |
| Lack of fuel/firewood | 0 | 79 | 79 | 0.019635 | 0 |
| Liking for meat | 0 | 78 | 78 | 0.019635 | 0 |
| Lack of knowledge | 0 | 64 | 64 | 0.019635 | 0 |
| Too many clients/users | 0 | 21 | 21 | 0.019635 | 0 |
| Hunger/too hungry | 0 | 17 | 17 | 0.019635 | 0 |
| Lack of cooking skills | 0 | 12 | 12 | 0.019635 | 0 |
| Insufficient meat cooking | 0 | 11 | 11 | 0.019635 | 0 |
| Negligence | 0 | 10 | 10 | 0.019635 | 0 |
| Lack of cooking utensils | 0 | 10 | 10 | 0.019635 | 0 |
| Laziness | 0 | 7 | 7 | 0.019635 | 0 |
| Alcohol abuse | 0 | 6 | 6 | 0.019635 | 0 |
| Profit maximisation | 0 | 4 | 4 | 0.019635 | 0 |
| Lack of water | 0 | 4 | 4 | 0.019635 | 0 |
| Lack of money/Economic reasons | 0 | 2 | 2 | 0.019635 | 0 |
| Customer retention reasons | 0 | 2 | 2 | 0.019635 | 0 |
| Household and neighbour conflicts | 0 | 2 | 2 | 0.019635 | 0 |
| Lack of stakeholder sensitisation | 0 | 2 | 2 | 0.019635 | 0 |
| Lack of centralised slaughter points | 0 | 1 | 1 | 0.019635 | 0 |
| Commercial mistrust | 0 | 1 | 1 | 0.019635 | 0 |
| Disability and weakness | 0 | 1 | 1 | 0.019635 | 0 |
| Poor quality pork | 0 | 1 | 1 | 0.019635 | 0 |
| Poor timing | 0 | 1 | 1 | 0.019635 | 0 |
| Traders avoid inspectors/fees/consquences | 0 | 1 | 1 | 0.019635 | 0 |
| Interference by other state/political actors | 0 | 1 | 1 | 0.019635 | 0 |
| Effect of cysticercosis not appreciated | 0 | 1 | 1 | 0.019635 | 0 |
| ill health (sickness) | 0 | 1 | 1 | 0.019635 | 0 |

**Table 5. Barrier to inspecting pork**

| **Barriers to inspecting pork** | **Weighted indegree** | **Weighted outdegree** | **Weighted degree** | **PageRank** | **Eigenvector centrality** |
| --- | --- | --- | --- | --- | --- |
| Lack of centralized slaughter points | 0 | 33 | 33 | 0.015591 | 0 |
| Lack of knowledge | 2 | 28 | 30 | 0.028832 | 0.039285 |
| Lack of applicable public laws/policies | 1 | 9 | 10 | 0.027845 | 0.114767 |
| Low staffing/Lack of inspectors/capacity gaps | 0 | 16 | 16 | 0.015591 | 0 |
| Resistance by some actors | 0 | 15 | 15 | 0.015591 | 0 |
| Insecurity | 1 | 2 | 3 | 0.022211 | 0.019643 |
| Interference by other state/political actors | 1 | 13 | 14 | 0.022211 | 0.019643 |
| Lack of inspection tool kits | 0 | 11 | 11 | 0.015591 | 0 |
| Corruption | 0 | 9 | 9 | 0.015591 | 0 |
| Lack of time | 0 | 8 | 8 | 0.015591 | 0 |
| Lack of money/Economic reasons | 0 | 7 | 7 | 0.015591 | 0 |
| Social; cultural and religious beliefs | 0 | 6 | 6 | 0.015591 | 0 |
| Traders avoid inspectors/fees/consequences | 0 | 6 | 6 | 0.015591 | 0 |
| Lack of stakeholder sensitization | 0 | 6 | 6 | 0.015591 | 0 |
| Lack of transport for inspectors | 0 | 5 | 5 | 0.015591 | 0 |
| Sick pigs are cheaper | 0 | 5 | 5 | 0.015591 | 0 |
| Inability to detect cysts | 0 | 5 | 5 | 0.015591 | 0 |
| lack of collaboration | 0 | 4 | 4 | 0.015591 | 0 |
| Liking for meat | 0 | 4 | 4 | 0.015591 | 0 |
| Negligence | 0 | 3 | 3 | 0.015591 | 0 |
| Poor mindset | 0 | 3 | 3 | 0.015591 | 0 |
| Laziness | 0 | 2 | 2 | 0.015591 | 0 |
| No compensation for condemned meat | 0 | 2 | 2 | 0.015591 | 0 |
| Alcohol abuse | 0 | 1 | 1 | 0.015591 | 0 |
| Poor quality pork | 0 | 1 | 1 | 0.015591 | 0 |
| Profit maximization | 0 | 1 | 1 | 0.015591 | 0 |
| Poor accessibility | 0 | 1 | 1 | 0.015591 | 0 |
| Inapparent infection | 0 | 1 | 1 | 0.015591 | 0 |
| Lack of inputs | 0 | 1 | 1 | 0.015591 | 0 |
| High cost of drugs | 0 | 1 | 1 | 0.015591 | 0 |
| Effect of cysticercosis not appreciated | 0 | 1 | 1 | 0.015591 | 0 |
| Lack of fuel/firewood | 0 | 1 | 1 | 0.015591 | 0 |
| ill health (sickness) | 0 | 1 | 1 | 0.015591 | 0 |

**Table 6. Barriers to handwashing, washing fruits and vegetables**

| **Barrier to handwashing and washing fruits and vegetables** | **Weighted indegree** | **Weighted outdegree** | **Weighted degree** | **PageRank** | **Eigenvector centrality** |
| --- | --- | --- | --- | --- | --- |
| Lack of knowledge | 1 | 90 | 91 | 0.028506 | 0.021425 |
| Lack of time | 1 | 34 | 35 | 0.030289 | 0.085247 |
| Liking for meat | 1 | 10 | 11 | 0.030289 | 0.085247 |
| Lack of water | 0 | 85 | 85 | 0.022208 | 0 |
| Lack of money/Economic reasons | 3 | 9 | 12 | 0.037953 | 0.04285 |
| Negligence | 0 | 47 | 47 | 0.022208 | 0 |
| Laziness | 0 | 46 | 46 | 0.022208 | 0 |
| Lack of handwashing/washing facilities | 0 | 42 | 42 | 0.022208 | 0 |
| Poor hygiene/sanitation practices | 0 | 28 | 28 | 0.022208 | 0 |
| Hunger/too hungry | 0 | 15 | 15 | 0.022208 | 0 |
| Social; cultural and religious beliefs | 0 | 14 | 14 | 0.022208 | 0 |
| Poor mindset | 0 | 7 | 7 | 0.022208 | 0 |
| Disability and weakness | 0 | 5 | 5 | 0.022208 | 0 |
| Lack of stakeholder sensitisation | 0 | 3 | 3 | 0.022208 | 0 |
| ill health (sickness) | 0 | 3 | 3 | 0.022208 | 0 |
| Resistence by some actors | 0 | 2 | 2 | 0.022208 | 0 |
| Travelling (Lack of public toilets) | 0 | 2 | 2 | 0.022208 | 0 |
| Deliberate spread of diseae | 0 | 1 | 1 | 0.022208 | 0 |
| Commercial mistrust | 0 | 1 | 1 | 0.022208 | 0 |
| Loss of food's aesthetic value | 0 | 1 | 1 | 0.022208 | 0 |
| Liking for fruits | 0 | 1 | 1 | 0.022208 | 0 |
| Lack of applicable public laws/policies | 0 | 1 | 1 | 0.022208 | 0 |
| Age (too young children) | 0 | 1 | 1 | 0.022208 | 0 |

**Table 7. Barriers to pig confinement**

| **Barrier** | **Weighted indegree** | **Weighted outdegree** | **Weighted degree** | **Eigenvector centrality** | **PageRank** |
| --- | --- | --- | --- | --- | --- |
| Lack of inputs | 1 | 34 | 35 | 0.042313 | 0.666667 |
| Lack of knowledge | 3 | 24 | 27 | 0.054297 | 0.666667 |
| Lack of money/Economic reasons | 4 | 16 | 20 | 0.067919 | 0.333333 |
| Lack of pigsty | 0 | 12 | 12 | 0.023068 | 0 |
| Lack of land | 0 | 6 | 6 | 0.023068 | 0 |
| Negligence | 0 | 6 | 6 | 0.023068 | 0 |
| Lack of applicable public laws/policies | 0 | 6 | 6 | 0.023068 | 0 |
| Resistance by some actors | 0 | 5 | 5 | 0.023068 | 0 |
| Free-range to improve growth rates | 0 | 4 | 4 | 0.023068 | 0 |
| Boar service | 0 | 2 | 2 | 0.023068 | 0 |
| Low input production system | 0 | 2 | 2 | 0.023068 | 0 |
| Lack of time | 0 | 2 | 2 | 0.023068 | 0 |
| Poor mindset | 0 | 2 | 2 | 0.023068 | 0 |
| Lack of construction materials | 0 | 3 | 3 | 0.023068 | 0.5 |
| Deliberate spread of disease | 0 | 1 | 1 | 0.023068 | 0 |
| Laziness | 0 | 1 | 1 | 0.023068 | 0 |
| Poor hygiene/sanitation practices | 0 | 1 | 1 | 0.023068 | 0 |
| Household and neighbor conflicts | 0 | 1 | 1 | 0.023068 | 0 |
| Weather | 0 | 1 | 1 | 0.023068 | 0 |
| Pigs as scavengers | 0 | 1 | 1 | 0.023068 | 0 |
| poorly constructed toilet or latrine | 0 | 1 | 1 | 0.023068 | 0 |

**Table 8. Barriers to toilet use as identified by community members**

| **Barrier to use of toilets** | **Weighted indegree** | **Weighted outdegree** | **Weighted degree** | **PageRank** | **Eigenvector centrality** |
| --- | --- | --- | --- | --- | --- |
| Lack of toilet | 0 | 60 | 60 | 0.020375 | 0 |
| Barrier | 342 | 0 | 342 | 0.470254 | 1 |
| Lack of knowledge | 0 | 55 | 55 | 0.020375 | 0 |
| Negligence | 0 | 26 | 26 | 0.020375 | 0 |
| Poor hygiene/sanitation practices | 0 | 23 | 23 | 0.020375 | 0 |
| Lack of money/Economic reasons | 0 | 21 | 21 | 0.020375 | 0 |
| ill health (sickness) | 0 | 20 | 20 | 0.020375 | 0 |
| Laziness | 0 | 19 | 19 | 0.020375 | 0 |
| Social; cultural and religious beliefs | 0 | 19 | 19 | 0.020375 | 0 |
| Disability and weakness | 0 | 15 | 15 | 0.020375 | 0 |
| Travelling (Lack of public toilets) | 0 | 12 | 12 | 0.020375 | 0 |
| Fear of toilet use | 0 | 9 | 9 | 0.020375 | 0 |
| Age (too young children) | 0 | 9 | 9 | 0.020375 | 0 |
| poorly constructed toilet or latrine | 0 | 9 | 9 | 0.020375 | 0 |
| Distance to the toilet | 0 | 8 | 8 | 0.020375 | 0 |
| Alcohol abuse | 0 | 7 | 7 | 0.020375 | 0 |
| Pregnancy | 0 | 6 | 6 | 0.020375 | 0 |
| Lack of land | 0 | 6 | 6 | 0.020375 | 0 |
| Poor soil texture | 0 | 4 | 4 | 0.020375 | 0 |
| Presence/closeness of bushes | 0 | 3 | 3 | 0.020375 | 0 |
| Weather | 0 | 3 | 3 | 0.020375 | 0 |
| Lack of time | 0 | 2 | 2 | 0.020375 | 0 |
| Household and neighbour conflicts | 0 | 2 | 2 | 0.020375 | 0 |
| To see own faeces | 0 | 1 | 1 | 0.020375 | 0 |
| Deliberate spread of diseae | 0 | 1 | 1 | 0.020375 | 0 |
| Fear to transmit/acquire infection | 0 | 1 | 1 | 0.020375 | 0 |
| Lack of water | 0 | 1 | 1 | 0.020375 | 0 |

**Table 9: Barriers to toilet use as identified by community leaders**

| **Barriers to toilet use** | | **Weighted indegree** | **Weighted outdegree** | **Weighted degree** | **PageRank** | **Eigenvector centrality** |
| --- | --- | --- | --- | --- | --- | --- |
| Lack of knowledge | 0 | | 5 | 5 | 0.056681 | 0 |
| Poor hygiene/sanitation practices | 0 | | 5 | 5 | 0.056681 | 0 |
| Lack of toilet | 0 | | 4 | 4 | 0.056681 | 0 |
| Laziness | 0 | | 2 | 2 | 0.056681 | 0 |
| Social; cultural and religious beliefs | 0 | | 2 | 2 | 0.056681 | 0 |
| Negligence | 0 | | 1 | 1 | 0.056681 | 0 |
| Poor mindset | 0 | | 1 | 1 | 0.056681 | 0 |
| Distance to the toilet | 0 | | 1 | 1 | 0.056681 | 0 |
| poorly constructed toilet or latrine | 0 | | 1 | 1 | 0.056681 | 0 |

**Table 10. Barriers to toilet use as identified by community health workers**

| **Barriers to toilet use** | **Weighted indegree** | **Weighted outdegree** | **Weighted degree** | **PageRank** | **Eigenvector centrality** | |
| --- | --- | --- | --- | --- | --- | --- |
| Social; cultural and religious beliefs | 0 | 18 | 18 | 0.034795 | 0 | |
| Lack of knowledge | 0 | 17 | 17 | 0.034795 | 0 | |
| Poor soil texture | 0 | 10 | 10 | 0.034795 | 0 | |
| Lack of money/Economic reasons | 0 | 10 | 10 | 0.034795 | 0 | |
| poorly constructed toilet or latrine | 0 | 5 | 5 | 0.034795 | 0 | |
| Lack of land | 0 | 4 | 4 | 0.034795 | 0 | |
| Lack of construction materials | 0 | 4 | 4 | 0.034795 | 0 | |
| Poor hygiene/sanitation practices | 0 | 3 | 3 | 0.034795 | 0 | |
| Lack of toilet | 0 | 2 | 2 | 0.034795 | 0 | |
| Poor mindset | 0 | 2 | 2 | 0.034795 | 0 | |
| Laziness | 0 | 1 | 1 | 0.034795 | 0 | |
| Disability and weakness | 0 | 1 | 1 | 0.034795 | 0 | |
| Population growth | 0 | 1 | 1 | 0.034795 | 0 | |
| Lack of inputs | 0 | 1 | 1 | 0.034795 | 0 | |
| Distance to the toilet | 0 | 1 | 1 | 0.034795 | 0 |  |
